# Improvement of Radioimmunotherapy Using Pretargeting

**DOI:** 10.3389/fonc.2013.00159

**Published:** 2013-06-20

**Authors:** Eric Frampas, Caroline Rousseau, Caroline Bodet-Milin, Jacques Barbet, Jean-Francois Chatal, Françoise Kraeber-Bodéré

**Affiliations:** ^1^Radiology Department, University Hospital, Nantes Cedex, France; ^*2*^Le Centre Régional de Recherche en Cancérologie Nantes/Angers, Centre national de la recherche scientifique, Université de Nantes, Nantes Cedex, France; ^*3*^Nuclear Medicine Department, Integrated Center of Oncology, Saint-Herblain, France; ^*4*^Nuclear Medicine Department, University Hospital, Nantes Cedex, France; ^*5*^Groupement d’Intérêt Public, Accelerator for Research in Radiochemistry and Oncology, Nantes Atlantic, Saint-Herblain, France

**Keywords:** radioimmunotherapy, pretargeting, bispecific antibody, CEA, avidin-biotin

## Abstract

During the past two decades, considerable research has been devoted to radionuclide therapy using radiolabeled monoclonal antibodies and receptor binding agents. Conventional radioimmunotherapy (RIT) is now an established and important tool in the treatment of hematologic malignancies such as Non-Hodgkin lymphoma. For solid malignancies, the efficacy of RIT has not been as successful due to lower radiosensitivity, difficult penetration of the antibody into the tumor, and potential excessive radiation to normal tissues. Innovative approaches have been developed in order to enhance tumor absorbed dose while limiting toxicity to overcome the different limitations due to the tumor and host characteristics. Pretargeting techniques (pRIT) are a promising approach that consists of decoupling the delivery of a tumor monoclonal antibody (mAb) from the delivery of the radionuclide. This results in a much higher tumor-to-normal tissue ratio and is favorable for therapy as well and imaging. This includes various strategies based on avidin/streptavidin-biotin, DNA-complementary DNA, and bispecific antibody-hapten bindings. pRIT continuously evolves with the investigation of new molecular constructs and the development of radiochemistry. Pharmacokinetics improve dosimetry depending on the radionuclides used (alpha, beta, and Auger emitters) with prediction of tumor response and host toxicities. New constructs such as the Dock and Lock technology allow production of a variety of mABs directed against tumor-associated antigens. Survival benefit has already been shown in medullary thyroid carcinoma. Improvement in delivery of radioactivity to tumors with these pretargeting procedures associated with reduced hematologic toxicity will become the next generation of RIT. The following review addresses actual technical and clinical considerations and future development of pRIT.

## Introduction

Conventional radioimmunotherapy (RIT) using an intravenously injected directly radiolabeled antibody has been extensively tested in preclinical and clinical studies. Its efficacy has been clearly documented in the most favorable clinical setting of disseminated small-size tumors especially those that are rapidly accessible to the injected antibody, such as bone marrow (Morschhauser et al., 2008). This efficacy has been limited in the situation of large tumor burden except for non-Hodgkin Lymphoma which is highly radiosensitive and thus needs a relatively small absorbed dose for an objective response. With these large tumors there is a considerable distance before the antibody molecule comes into contact with cancer cells. Some physiological barriers prevent the rapid diffusion of an antibody into the tumor. After reaching it the antibody molecule first encounters the antigen present at the surface of tumor cells in the perivascular space. A high binding affinity of the antibody can impede it from migrating deeply into the tumor whereas a low binding affinity allows a deeper penetration into the tumor (Sharkey and Goldenberg, 2005). After injection it takes 2–3 days before a maximum level of antibody concentration is reached within the tumor. Finally, in general only a very small fraction of the injected activity will localize to the tumor, which explains the modest response rate that is usually observed.

As optimal tumor targeting requires a high tumor uptake and a low retention of radioactivity in normal tissues, especially in the blood, the situation is not favorable for large tumors over 3–4 cm in diameter. One way to improve this situation is to hasten antibody blood clearance by reducing the molecular size. Molecular engineering has allowed production of antibody forms with varied valencies and molecular sizes. One problem with smaller sized antibodies or antibody fragments is a faster clearance from the blood which is favorable for normal tissue toxicity, but also results in a low tumor uptake and retention with low tumor absorbed dose and no therapeutic efficacy.

Thus it appears that for unique or multiple macroscopic tumor targets larger than 3–4 cm in diameter, RIT with directly labeled antibodies cannot be therapeutically efficient. At best it can bring a useful contribution when used in combination with chemotherapy. This is why there is a critical need for innovative approaches to allow enhanced tumor absorbed doses whilst limiting hematologic toxicity. Pretargeting techniques can at least partially solve this problem.

## Pretargeted RIT Techniques

The principle of pretargeted RIT consists of decoupling the delivery of a tumor specific antibody from the delivery of the radionuclide. In the first step a large saturating dose of the unlabeled antibody is injected and distributes throughout the body. The antibody molecule slowly binds to the tumor cells while clearing from other tissues. In the second step, at a later time of 2–4 days, when the concentration of the antibody in the tumor is at its peak and much higher than in normal organs, a radioactive small molecule is injected and rapidly reaches the tumor where it specifically binds to the pre-localized antibody. By comparison to the method of directly labeled antibody it results a much higher tumor-to-normal tissue ratio which is favorable for therapy.

### Methods based on avidin/streptavidin-biotin binding

Several pretargeting methods have been developed including two in clinical studies: one of them was based on the four binding sites and the very high affinity (10^−15^ M) of avidin for biotin (Hnatowich et al., 1987). In a first approach streptavidin is conjugated to an antibody molecule and intravenously injected. A few days later, radiolabeled biotin is injected and rapidly binds to tumor-localized streptavidin. Due to the large size of the streptavidin-antibody conjugate that results in a long residence time in the blood it is necessary to remove excess streptavidin-antibody conjugate from the blood using a clearing agent before injection of the radiolabeled biotin. In a second approach, biotin is conjugated to the antibody molecule. One or two days later, avidin which is glycosylated and rapidly cleared by the liver, is injected and removes biotin-antibody conjugate from the blood. Thirty minutes later streptavidin, which is not glycosylated and thus not cleared by the liver, is injected and after reaching the tumor binds strongly to the pre-localized biotin-antibody conjugate resulting in a high tumor-to-normal tissue ratio similar to the first approach.

Several antibodies have been conjugated with streptavidin and clinically tested (Knox et al., 2000; Shen et al., 2005). Interestingly, a 10-fold improvement of tumor-to-normal tissue ratios was observed with regard to the same directly radiolabeled antibodies confirming the interest of this approach. Using the three-step method with a biotin-antibody conjugate followed by avidin and streptavidin and then radiolabeled biotin, several clinical trials have been performed in Milan, Italy in patients with high-grade gliomas (Paganelli et al., 1998; Cremonesi et al., 1999; Grana et al., 2002). In comparison to a directly radiolabeled antibody, the three-step method allowed the injection of an activity of ^90^Y-biotin five times higher due to a much lower blood radioactive concentration. A total of 85 patients have been treated in 2 successive phase I and II studies. Quite interestingly, after a long follow-up life expectancy was longer than in the control group. Survival time was 33.5 months as compared with only 8 months for the control group (Grana et al., 2002).

### Method based on DNA-complementary DNA binding

The rationale for choosing this binding system is based on the high affinity of the interaction and non-immunogenicity of the oligonucleotides. However, the suitability of the native phosphodiester DNA as an effector is limited by its susceptibility to nuclease hydrolysis. Morpholino oligomers (MORFs), are synthetic DNA analogs that have been reported to be water soluble, stable to nucleases, and highly specific for its complementary MORF (Liu et al., 2002). Several preclinical studies have been performed with ^188^Re (Liu et al., 2010) and ^90^Y (Liu et al., 2011) and tended to confirm the interest of this pretargeting technique, with high tumor-to-normal tissue ratios but no clinical study has been implemented yet.

### Method based on bispecific antibody-hapten binding

This alternative method initially called “Affinity Enhancement System (AES)” was first designed and developed in France and later optimized in the USA. It consists of firstly injecting an unlabeled bispecific antibody (BsmAb) made of an equimolecular amount of a Fab’ fragment of an anti-carcinoembryonic antigen (CEA) antibody and a Fab’ fragment of an anti-DTPA-indium antibody. A few days later a radiolabeled bivalent hapten that quickly binds to the pre-localized BsmAb is injected and. In this system, the affinity of the hapten is limited but the bivalent hapten binds avidly to the BsmAb bound at the surface of tumor cells whereas the BsmAb-hapten complexes can dissociate in the blood with clearance of excess hapten through the kidneys.

The advantage of this pretargeting system with regard to directly radiolabeled antibodies was first documented preclinically in CEA-positive-human colorectal tumors (Gautherot et al., 1997). Biodistribution and therapeutic efficacy with the two-step method were compared with that of a directly labeled antibody using iodine-125 or iodine-131. With the directly labeled antibody maximal tumor uptake was observed late at 2 days and was higher than that of the two-step technique which showed a maximal tumor uptake at 1 h. Meanwhile, the directly labeled antibody cleared slowly from the blood by comparison with the radiolabeled hapten which cleared very rapidly. As a result very high tumor-to-blood ratios were achieved quickly with the two-step method. Consequently, for therapeutic application, a higher activity of iodine-131 was injected with the two-step method (111 MBq) compared to only 12 MBq with the directly labeled antibody, resulting in an equivalent hematologic toxicity in both groups. Due to the much higher injected activity with the radiolabeled hapten, therapeutic efficacy was much better with the two-step method with a tumor growth inhibition sustained over 150 days compared to the directly labeled antibody which induced only a growth delay of 53 days.

The results of this preclinical study were then confirmed by a clinical study in 11 patients with primary colorectal cancer (Le Doussal et al., 1993). These patients were injected with 1–10 mg of an anti-CEA/anti-DTPA BsmAb and 2–8 days later with an ^111^In-labeled DTPA dimer (222 MBq). The tumor was surgically resected 1–4 days after the last injection. The biodistribution results were compared with those obtained in six patients with similar clinical status and injected with directly labeled ^111^In-labeled anti-CEA F(ab’)_2._ Tumor uptake measured from resected tumors using the two-step approach was 1.8–17.5% injected dose/kg and not significantly lower than that found in the six patients injected with directly labeled antibody (5.5–30.2% injected dose/kg). Interestingly however, tumor-to-blood and tumor-to-liver ratios were significantly improved with the two-step method in comparison with the one-step method (7.8 versus 4.2 for blood and 2.8 versus 0.8 for liver).

At this time the next consideration was what was the most appropriate clinical setting to clearly demonstrate the superiority of pretargeted RIT over conventional RIT with directly radiolabeled antibody. The final choice was medullary thyroid carcinoma (MTC) despite the low frequency of this type of cancer. Indeed this type of cancer is known to be well-vascularized and expresses a high density of CEA at the cell surface. Moreover, some specific biomarkers, namely calcitonin (Ct) and CEA, allow the evolution of the disease and the response to treatment to be monitored. Pretargeting studies in this setting started at the beginning of the 1990s and lasted more than 15 years.

## AES Pretargeted RIT in Medullary Thyroid Carcinoma

### Conventional treatment and prognosis of MTC

Medullary thyroid carcinoma represents less than 10% of all thyroid carcinoma and occurs both as a familial and a sporadic disease (Tubiana et al., 1968). Total thyroidectomy with lymph node dissection is the primary treatment. After surgery, serum Ct is not detectable in more than 60% of patients without lymph node involvement, versus less than 20% of patients with lymph node spread (Machens et al., 2005; Fialkowski et al., 2008). As with other neuroendocrine tumors, prognosis of MTC is very heterogeneous and determination of prognostic indicators appears relevant (Byar et al., 1979; Tisell et al., 2003; Ito et al., 2005; Machens et al., 2005; Elisei et al., 2008; Fialkowski et al., 2008). In metastatic disease, cytoreductive therapeutic options are limited. Targeted therapy can be applied in progressive metastatic patients. Recently, disease stabilization has been reported with targeted therapy using multikinase inhibitors (MKIs) in advanced or metastatic MTC, and vandetanib has been approved in the USA (de Groot et al., 2007; Schlumberger et al., 2009; Lam et al., 2010; Wells et al., 2010, 2012). Prognosis of metastatic MTC varies from long- to short-term survival and highly reliable prognostic factors are needed for early detection of high-risk patients who require treatment, whereas low-risk patients warrant a “watch-and-wait” approach. The identification of predictive markers of response and survival appears very important in selecting patients most likely to benefit from a systemic therapy and to avoid exposing patients who are unlikely to respond or who have a long life expectancy from possible treatment-related adverse events and associated costs.

Among the various prognostic parameters that could identify high- and low-risk groups, advanced age, stage of the disease, the EORTC prognostic scoring system, and association with multiple endocrine neoplasia (MEN) 2B are commonly accepted as prognostic factors (Byar et al., 1979; Tisell et al., 2003; Ito et al., 2005; Fialkowski et al., 2008). Moreover, mutations in the RET oncogene are associated with lower survival rates (Elisei et al., 2008). The Cdc25B phosphatase has also been shown as a new indicator of aggressive MTC (Ito et al., 2005). Tumor aggressiveness measured by Ki67 expression has been described as another prognostic factor (Tisell et al., 2003). By monitoring serum Ct and CEA concentration kinetics and calculating biomarker doubling-times (DT), Barbet et al. (2005) demonstrated that Ct DT was an independent predictor of survival, with a high predictive value, in patients with measurable serum Ct, even after repeated surgery. In this study, all 41 patients with Ct DT>2 years were still alive at the end of the study, 2.9–29.5 years after initial surgery. Eight patients (67%) with DT between 6 months and 2 years died of the disease 40–189 months after surgery, and all 12 patients with Ct DT<6 months died of the disease 6 months–13.3 years after initial surgery. Laure Giraudet et al. (2008) confirmed the prognostic value of biomarker DT in a series of 55 consecutive patients.

### Immunodetection with pretargeting

Before assessing the efficacy in a therapeutic application, pretargeting was evaluated using scintigraphic imaging with anti-CEA × anti-DTPA-indium BsmAb and 111In-labeled bivalent DTPA hapten in preclinical and clinical studies (Vuillez et al., 1992; Peltier et al., 1993; Barbet et al., 1998; Hosono et al., 1998). In preclinical MTC models, pretargeting using AES method has demonstrated a more favorable therapeutic index than directly labeled anti-CEA mAb (Hosono et al., 1998). In the first clinical study performed in eight patients, immunoscintigraphy visualized all known tumors and detected previously unknown tumor sites (US and CT negative) in the neck and the liver (Peltier et al., 1993). There were no false-positive results. Immunoscintigraphy was completed by radioimmunoguided surgery with the help of a hand-held gamma probe. This technique allowed small tumors not detected by the surgeon to be localized but failed to detect two small lesions (1 × 1 cm) corresponding to fibrosis with infiltrated microscopic cancer. This first study was followed by a larger study performed on 44 patients with elevated Ct serum levels after resection of the primary MTC. Immunoscintigraphy was performed 2, 5, and 24 h after hapten injection and, when necessary, at longer time intervals. When available, a hand-held gamma probe was used during surgery. Fifteen patients had known tumor sites before immunoscintigraphy. Tumors were imaged in 12 (80%) of these patients, including three with liver metastases. Five unknown tumor sites were detected. For the 29 patients with occult disease, immunoscintigraphy detected high-activity uptake sites in 21 patients (72%), including 5 in the liver. Twelve were confirmed by surgery, one by guided morphologic imaging, and one by venous catheterization. Radioimmunoguided surgery was used on 14 patients. It was considered helpful by the surgeon in 12 patients, including 4 patients where it allowed the resection of small, non-palpable nor visible, tumor-involved lymph nodes. This short imaging study confirmed the advantage of pretargeting and provided an incentive to proceed to go to the next step of RIT.

### RIT in metastatic MTC

A first phase I/II study was conducted in patients with metastatic MTC with the F(ab’)_2_ fragment of the anti-CEA mAb MN-14 labeled with iodine-131 (Juweid et al., 1999). Fifteen patients were enrolled in this study. Myelosuppression was the only significant treatment-related dose-limiting toxicity. Human anti-mouse antibodies (HAMA) developed in eight patients. Seven patients had a median of 55% reduction of serum biomarkers. One patient showed a dramatic improvement in the mass effect on the airways caused by three tumor lesions in the neck, with a 45% reduction of overall tumor burden. The disease has continued to be radiologically stable in 11 of 12 assessable patients for periods ranging from 3+ to 26+ months.

A second phase I clinical study was performed using the same radiolabeled mAb to determine the toxicity and therapeutic potential of high-dose ^131^I-MN-14 F(ab)_2_ combined with autologous hematopoietic stem cell rescue in patients with rapidly progressing metastatic MTC (Juweid et al., 2000). Twelve patients were enrolled in this study. Starting doses were 900 cGy to the kidney and no more than 1200 cGy to the lung and liver, with dose increments of 300 cGy until the maximum tolerable dose is determined. Post-RAIT scintigraphies showed a tumor targeting in all patients. Autologous hematopoietic stem cells were given to all patients 1–2 weeks after therapy. Except for the instance of grade 3 gastrointestinal toxicity, non-hematologic toxicity was relatively mild, with only grade 1 or 2 toxicity observed in nine patients. No renal toxicity was seen. Of the 12 patients, 1 had partial remission for 1 year, another had a minor response for 3 months, and 10 had stabilization of disease lasting between 1 and 16 months.

**Figure 1:**
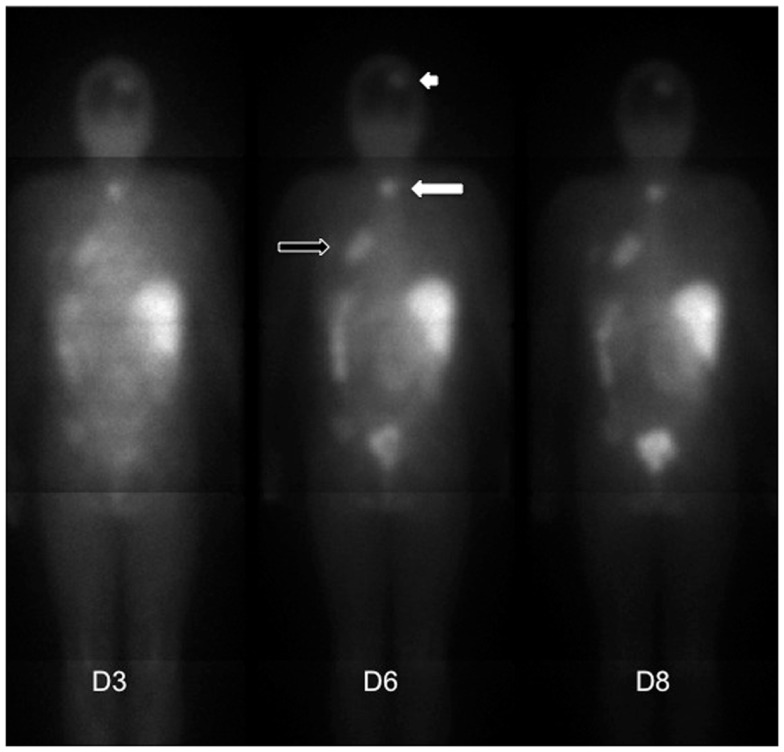
**Immunoscintigraphies (posterior view) recorded in a patient with a metastatic CEA-positive small cells lung cancer**. This patient was injected with the anti-CEA hMN14x734 bispecific antibody and a therapeutic activity of ^131^I-di-DTPA-indium. The immunoscintigraphies recorded 3, 6, and 8 days after hapten injection showed a good uptake in lung (black arrow), mediastinum (white arrow), and brain (white arrowhead) metastases.

A phase I/II clinical trial was started in 1996 to evaluate toxicity, pharmacokinetics, dosimetry, and anti-tumor activity of pRAIT using murine anti-CEA × anti-DTPA BsMAb F6x734 and a bivalent indium-DTPA hapten labeled with iodine-131, in 26 patients with metastatic MTC (Kraeber-Bodéré et al., 1999a). A good tumor targeting was observed in the majority of patients, with a high bone marrow uptake reflecting the high frequency of bone marrow involvement confirmed by MRI (Mirallié et al., 2005). Dose-limiting toxicity was hematological and maximum tolerated activity was estimated at 1.8 GBq/m^2^ in the group of patients with suspected bone marrow involvement. Some tumor responses were observed, mainly in patients with a small tumor burden and after repeated courses of pRAIT. Because of a relatively high hematological toxicity and frequent immune responses, the chimeric anti-CEA × anti-DTPA hMN14x734 BsMAb was developed. A prospective phase I optimization study was performed in 34 patients with CEA-expressing tumors to determine optimal BsMAb dose, hapten activity, and pretargeting interval (Kraeber-Bodéré et al., 1999b, 2006). A BsMAb dose of 40 mg/m^2^ with a pretargeting interval of 5 days appeared to be a good compromise between toxicity and efficacy. HAMA elevation was observed in 8% of patients and HAHA (human anti-human antibody) in 33%. Figure 1 shows the good tumor uptake observed in a patient with a metastatic relapsing CEA-positive small cells lung cancer after injection of a therapeutic activity of indium-DTPA hapten labeled with iodine-131.

In 2006, Chatal et al. (2006) published a retrospective analysis of survival of the series of MTC patients involved in the two phase I/II pRIT clinical trials, comparing the survival of 29 patients given pRIT with that of 39 contemporaneous untreated patients for whom data were collected by the French Endocrine Tumor Group (GTE). A second objective was to examine whether post-pRIT variations of Ct DT could be used as a surrogate marker for survival by comparing, among treated patients, the survival of biological responders and non-responders, defining a responder as showing at least a 100% increase in Ct DT. The patients were stratified in risk-groups according to Ct DT, and patients with Ct DT<2 years were considered as high-risk patients. This study showed that overall survival (OS) was significantly longer in high-risk treated patients than in high-risk untreated patients (median OS, 110 versus 61 months; *P* < 0.030). Treated patients with bone/bone marrow disease had a longer survival than patients without such involvement (10-year OS of 83 versus 14%; *P* < 0.023). Toxicity was mainly hematological and related to bone marrow involvement. Patients with grade 4 thrombocytopenia received platelet infusions and those with grade 4 leucopenia received G-CSF injections. No kidney toxicity was observed after pRIT.

**Figure 2:**
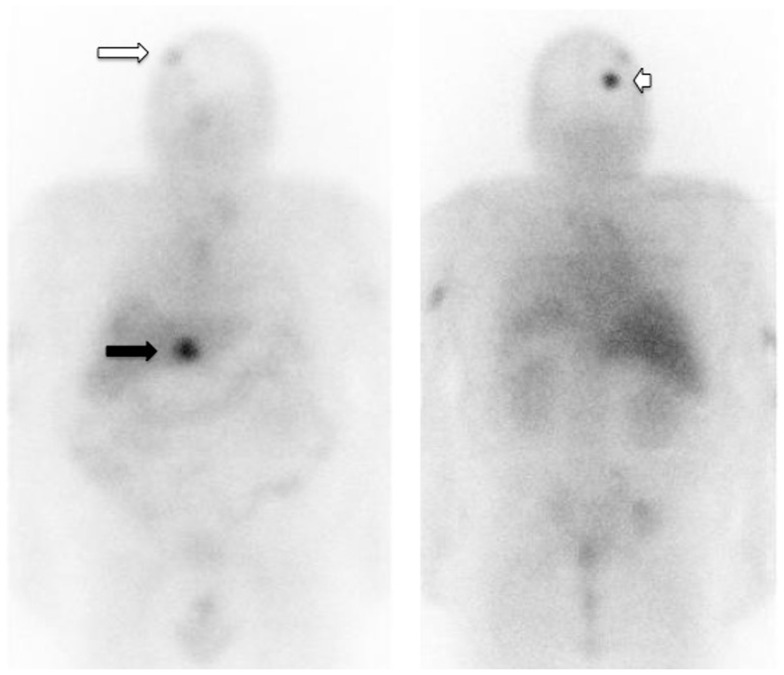
**Immunoscintigraphy (anterior and posterior views) recorded in a patient with metastatic medullary thyroid carcinoma**. This patient was injected with the anti-CEA hMN14x734 bispecific antibody and a therapeutic activity of 131I-di-DTPA-indium. The immunoscintigraphy recorded 4 days after hapten injection showed a good tumor uptake in liver (black arrow), bone (white arrow), and brain (white arrowhead) metastases.

Following the encouraging results obtained in the two phase I/II studies, a prospective phase II multicenter pRIT trial was undertaken in progressive MTC patients with Ct DT shorter than 5 years. From 2004 to 2008, 42 MTC patients were treated with 40 mg/m^2^ of hMN-14xm734 and 1.8 GBq/m^2^ ^131^I-di-DTPA-indium bivalent hapten 4–6 days later (Salaun et al., 2012). Patients were stratified in risk-groups according minimal DT (lowest value between Ct- and CEA-DT) as follows: high-risk patients with minimal DT lower than 6 months, intermediate-risk with DT between 6 months and 2 years, and low-risk with DT longer than 2 years. Disease control according RECIST criteria (objective response + stabilization) was observed in 32 patients (76.2%), including a durable complete response of at least 40 months in 1 patient (2.4%) and durable stable disease (≥6 months) in 31 patients (73.8%). Tumor uptake assessed by post-pRIT immunoscintigraphy was a significant predictor of response. Figure 2 shows the good tumor uptake in a metastatic MTC patient. Subacute toxicity was mainly hematological, requiring careful post-RAIT blood monitoring. Pre-RAIT biomarker DT and impact on DT after pRIT were predictors of OS, confirming the value of serum biomarkers in selecting patients and monitoring therapy.

Radioimmunotherapy could also be applied in a multimodality strategy, especially in MTC patients with measurable metastatic lesions. A synergistic effect was observed in MTC animal models using a combination of RAIT with paclitaxel (Kraeber-Bodéré et al., 2002). Improvement of tumor response was also demonstrated using a combination of RAIT with anti-angiogenic agents such as thalidomide, CBOP11 (cyclopeptidic vascular endothelial growth inhibitor), or bevacizumab (Kraeber-Bodéré et al., 2010; Salaun et al., 2010). Pretreatment with bevacizumab improved RIT efficacy, with similar toxicity as compared to RIT alone. Pretreatment by CBOP11 or thalidomide sensitized larger tumors (>300 mm^3^), with increased leukopenia but not thrombocytopenia. An increase of the anti-tumor effect observed using the anti-angiogenic drug combined with RIT was correlated with a decrease of blood vessels shown by von Willebrand immunostaining. These combinations could be assessed in clinical trials in MTC patients with progressive and measurable tumor masses.

## Future Developments of Pretargeted RIT

### Method based on bispecific antibody-hapten binding

The two radionuclides usually used for RIT are yttrium-90 and lutetium-177 with a DOTA (1,4,7,10-tetraazacyclododecane-1,4,7,10-tetraacetic acid) chelating agent used for a stable binding. This chelating agent is not adapted to anti-DTPA(In) antibody which has been used up to now. Indeed, to ensure a high affinity binding to this antibody it is necessary to load DTPA with indium and then to load DOTA for a good stability binding with yttrium-90 or lutetium-177. Consequently it was necessary to develop another hapten-binding system. An anti-hapten antibody directed to a derivative of histamine-succinyl-glycine (HSG) with a high affinity was produced. Subsequently, a di-HSG-peptide was prepared containing a single DOTA and allowed accommodation of a number of radionuclides for imaging and therapeutic applications including ^68^Ga, ^124^I, ^18^F, ^90^Y, and ^177^Lu (Sharkey et al., 2010).

Concerning the optimal type of construct including the anti-CEA and anti-HSG antibodies it appeared that the trivalent form with two valencies for CEA and one for HSG had the highest targeting sensitivity. However, such an agent could not be efficiently produced with acceptable yields. That problem led to the design and development of an innovative and sophisticated system called Dock and Lock (Rossi et al., 2006). Finally, a trivalent bispecific structure referred to as TF2 and prepared using this Dock and Lock method was composed of three stably linked Fab fragments directed to CEA and a HSG peptide. The HSG peptide allows facile and stable labeling with different radiometals, such as ^177^Lu or ^90^Y, having favorable physical features that could improve pRIT efficacy (Goldenberg et al., 2008; Schoffelen et al., 2010a).

Pretargeting utilizes separate administrations of these two agents, and introduces new complexities concerning administration (molar doses of the agents, time interval between injections and bispecific antibody to peptide ratios) in order to optimize the treatment protocol. TF2 rapidly decreases in blood to less than 0.1% ID/g 24 h following injection. With a standard interval time of 24 h between the two agents, rapid tumor uptakes of 15.5% ID/g have been reported 1 h following the injection of the radiolabeled peptide in a hepatic metastases model of colonic carcinoma in mice, slowly decreasing to 4.6% ID/g at 48 h. In contrast, normal tissue uptakes were particularly low with maximal uptakes of 0.73% DI/g in blood and 2.38% ID/g in the kidneys leading to high tumor to non-tumor ratios, especially in the blood, as compared to direct RIT. Dosimetry and toxicity may be planned from models and pharmacokinetics, and the HSG peptide allows labeling with different radionuclides. As expected, due to the rapid clearance of the peptide, short-lived ^211^At was predicted to deliver the highest absorbed dose to the tumors, kidney being dose limiting. Other nuclides such as ^90^Y and ^213^Bi would also deliver highly absorbed doses to the tumors with acceptable absorbed dose to the kidneys. Beta emitters such as ^90^Y could be effective against larger tumors with an estimated absorbed dose of 4.2 Gy for subcutaneous tumors of 6 mm, with a favorable kidney dosimetry. A major point is that no significant toxicity to the bone marrow should be expected owing to the low activity in blood. (Frampas et al., 2011a,b).

Some clinical trials are ongoing using the trivalent bispecific anti-CEA and anti-HSG antibody TF2 and the ^177^Lu-DOTA-di-HSG-peptide IMP-288 in patients with colorectal cancer and small cell lung cancer.

The new generation AES products have a rapid distribution, suggesting benefit of labeling with short half-live PET emitters such as ^68^Ga or ^18^F, to develop a highly sensitive and specific immuno-PET method in CEA-expressing tumors. Immuno-PET and RIT could be applied in the context of a theranostic strategy (McBride et al., 2006, 2009; Karacay et al., 2011). Schoffelen et al. (2010b) developed procedures for radiolabeling a small hapten-peptide (1451 Da) with ^68^Ga or ^18^F to compare their specificity with ^18^F-FDG for detecting tumors using a pretargeting procedure in animal models of CEA-positive-human colonic tumor and a CEA-negative tumor, or a local inflammation. The intravenous injection in mice of anti-CEA × anti-hapten BsMAb was followed 16 h later by the injection of 5 MBq of ^68^Ga- or ^18^F-labeled hapten peptides. Within 1 h, the CEA-positive tumor showed high and specific targeting of ^68^Ga-IMP-288 (10.7 ± 3.6% ID/g tumor uptake) and very low uptake in normal tissues (tumor-to-blood ratio of 69.9 ± 32.3), compared to the CEA-negative tumor and inflamed muscle (lower than 1.0% ID/g). Results were similar with ^18^F-labeled IMP-449. Contrary, ^18^F-FDG lacked in specificity as it localized efficiently to the tumor but also in the inflamed muscle (7.42 ± 0.20 and 4.07 ± 1.13% ID/g, respectively) and in several normal tissues. Pretargeted Immuno-PET appears as a highly sensitive and specific imaging method, well fitted with short-lived radionuclides, more specific than ^18^F-FDG-PET. Two immuno-PET clinical studies using new generation AES reagents and ^68^Ga started in 2012 in France, with the aim to demonstrate the feasibility of the method in CEA-positive MTC and breast carcinoma patients and to optimize the pretargeting parameters.

### Method based on DNA-complementary DNA binding

To improve the therapeutic index using the morpholino phosphorodiamidate oligomer (MORF)/complement MORF (cMORF) pretargeting system the CC49 antibody directed to TAG-72 tumor antigen has been conjugated with both biotin and MORF in tumor-bearing mice (Liu et al., 2010). This directly labeled antibody has been extensively tested in preclinical and clinical studies. One day later avidin was injected and led to a decrease of radioactive concentration in blood but not in the tumor resulting in a highly improved therapeutic index. This result should be confirmed in clinical studies.

## Conclusion

There is no doubt that some advantages of pretargeted RIT have been clearly documented both in preclinical and clinical studies with regards to directly radiolabeled antibodies. This allows a significant increase in the tumor-to-normal tissue ratios, especially tumor-to-blood ratios, and subsequently allows a higher radioactivity level to be injected without impairing hematologic toxicity. Survival benefit has been shown using the two main pretargeting models. With the three-step pretargeting using biotinylated anti-tenascin antibody, avidin/streptavidin and ^90^Y-biotin, encouraging results have been obtained in patients with high-grade glioma (Paganelli et al., 2001). With the bispecific antibody-hapten pretargeting model a survival gain has also been documented in patients with rapidly progressing metastatic MTC (Chatal et al., 2006). Interestingly there were few objective responses but a long-term stabilization up to more than 10 years was observed in patients who progressed rapidly before RIT.

We can wonder what would be the best clinical indication for pretargeted RIT. Conventional RIT with directly labeled antibodies has proved to be efficient in the setting of disseminated microscopic disease with substantial gain in survival (Liersch et al., 2005, 2007; Morschhauser et al., 2008). In this situation of microscopic clusters of malignant cells located in bone marrow and thus rapidly accessible to intravenously injected antibody the potential interest of pretargeting is not obvious. Otherwise large tumors, more than 3–4 cm in diameter, are badly vascularized with necrotic regions which can prevent both directly labeled antibodies or unlabeled immunoconjugate from reaching and binding to tumor cells. Consequently the most appropriate situation could be intermediate tumor sizes in the range of 1–3 cm in diameter where tumor necrosis is still limited. In this situation the distribution of antibodies (labeled and unlabeled) could be roughly homogenous 2 or 3 days after injection and the pretargeting technique would allow injecting a higher activity level without impairing hematological toxicity. The ongoing clinical trials with the Dock and Lock technique are awaited before ascertaining the real future role of pretargeted immuno-Pet and RIT.

## Conflict of Interest Statement

The authors declare that the research was conducted in the absence of any commercial or financial relationships that could be construed as a potential conflict of interest.

## References

[B1] Barbet J. Campion L. Kraeber-Bodéré F. Chatal J.-F. (2005). Prognostic impact of serum calcitonin and carcinoembryonic antigen doubling-times in patients with medullary thyroid carcinoma. J. Clin. Endocrinol. Metab. 90, 6077–6084 10.1210/jc.2005-0044 16091497

[B2] Barbet J. Peltier P. Bardet S. Vuillez J. P. Bachelot I. Denet S. (1998). Radioimmunodetection of medullary thyroid carcinoma using indium-111 bivalent hapten and anti-CEA x anti-DTPA-indium bispecific antibody. J. Nucl. Med. 39, 1172–1178 9669389

[B3] Byar D. P. Green S. B. Dor P. Williams E. D. Colon J. van Gilse H. A. (1979). A prognostic index for thyroid carcinoma. A study of the E.O.R.T.C. Thyroid Cancer Cooperative Group. Eur. J. Cancer 15, 1033–1041 510341 10.1016/0014-2964(79)90291-3

[B4] Chatal J.-F. Campion L. Kraeber-Bodéré F. Bardet S. Vuillez J.-P. Charbonnel B. (2006). Survival improvement in patients with medullary thyroid carcinoma who undergo pretargeted anti-carcinoembryonic-antigen radioimmunotherapy: a collaborative study with the French Endocrine Tumor Group. J. Clin. Oncol. 24, 1705–1711 10.1200/JCO.2005.04.4917 16549819

[B5] Cremonesi M. Ferrari M. Chinol M. Stabin M. G. Grana C. Prisco G. (1999). Three-step radioimmunotherapy with yttrium-90 biotin: dosimetry and pharmacokinetics in cancer patients. Eur. J. Nucl. Med. 26, 110–120 10.1007/s002590050366 9933344

[B6] de Groot J. W. B. Zonnenberg B. A. van Ufford-Mannesse P. Q. de Vries M. M. Links T. P. Lips C. J. M. (2007). A phase II trial of imatinib therapy for metastatic medullary thyroid carcinoma. J. Clin. Endocrinol. Metab. 92, 3466–3469 10.1210/jc.2007-0649 17579194

[B7] Elisei R. Cosci B. Romei C. Bottici V. Renzini G. Molinaro E. (2008). Prognostic significance of somatic RET oncogene mutations in sporadic medullary thyroid cancer: a 10-year follow-up study. J. Clin. Endocrinol. Metab. 93, 682–687 10.1210/jc.2007-1714 18073307

[B8] Fialkowski E. DeBenedetti M. Moley J. (2008). Long-term outcome of reoperations for medullary thyroid carcinoma. World J. Surg. 32, 754–765 10.1007/s00268-007-9317-7 18188643

[B9] Frampas E. Maurel C. Remaud-Le Saëc P. Mauxion T. Faivre-Chauvet A. Davodeau F. (2011a). Pretargeted radioimmunotherapy of colorectal cancer metastases: models and pharmacokinetics predict influence of the physical and radiochemical properties of the radionuclide. Eur. J. Nucl. Med. Mol. Imaging 38, 2153–2164 10.1007/s00259-011-1903-0 21858527

[B10] Frampas E. Maurel C. Thedrez P. Remaud-Le Saëc P. Faivre-Chauvet A. Barbet J. (2011b). The intraportal injection model for liver metastasis: advantages of associated bioluminescence to assess tumor growth and influences on tumor uptake of radiolabeled anti-carcinoembryonic antigen antibody. Nucl. Med. Commun. 32, 147–154 10.1097/MNM.0b013e328341b268 21116205

[B11] Gautherot E. Bouhou J. Le Doussal J. M. Manetti C. Martin M. Rouvier E. (1997). Therapy for colon carcinoma xenografts with bispecific antibody-targeted, iodine-131-labeled bivalent hapten. Cancer 80, 2618–2623 10.1002/(SICI)1097-0142(19971215)80:12+<2618::AID-CNCR37>3.3.CO;2-P 9406716

[B12] Goldenberg D. M. Rossi E. A. Sharkey R. M. McBride W. J. Chang C.-H. (2008). Multifunctional antibodies by the Dock-and-Lock method for improved cancer imaging and therapy by pretargeting. J. Nucl. Med. 49, 158–163 10.2967/jnumed.107.046185 18077530

[B13] Grana C. Chinol M. Robertson C. Mazzetta C. Bartolomei M. De Cicco C. (2002). Pretargeted adjuvant radioimmunotherapy with yttrium-90-biotin in malignant glioma patients: a pilot study. Br. J. Cancer 86, 207–212 10.1038/sj.bjc.6600047 11870507 PMC2375191

[B14] Hnatowich D. J. Virzi F. Rusckowski M. (1987). Investigations of avidin and biotin for imaging applications. J. Nucl. Med. 28, 1294–1302 3612292

[B15] Hosono M. Hosono M. N. Kraeber-Bodéré F. Devys A. Thédrez P. Fiche M. (1998). Biodistribution and dosimetric study in medullary thyroid cancer xenograft using bispecific antibody and iodine-125-labeled bivalent hapten. J. Nucl. Med. 39, 1608–1613 9744353

[B16] Ito Y. Yoshida H. Tomoda C. Uruno T. Takamura Y. Miya A. (2005). Expression of cdc25B and cdc25A in medullary thyroid carcinoma: cdc25B expression level predicts a poor prognosis. Cancer Lett. 229, 291–297 10.1016/j.canlet.2005.06.040 16095809

[B17] Juweid M. E. Hajjar G. Stein R. Sharkey R. M. Herskovic T. Swayne L. C. (2000). Initial experience with high-dose radioimmunotherapy of metastatic medullary thyroid cancer using 131I-MN-14 F(ab)2 anti-carcinoembryonic antigen MAb and AHSCR. J. Nucl. Med. 41, 93–103 10647610

[B18] Juweid M. E. Hajjar G. Swayne L. C. Sharkey R. M. Suleiman S. Herskovic T. (1999). Phase I/II trial of (131)I-MN-14F(ab)2 anti-carcinoembryonic antigen monoclonal antibody in the treatment of patients with metastatic medullary thyroid carcinoma. Cancer 85, 1828–1842 10.1002/(SICI)1097-0142(19990415)85:8<1828::AID-CNCR25<;3.0.CO;2-H 10223579

[B19] Karacay H. Sharkey R. M. McBride W. J. Rossi E. A. Chang C.-H. Goldenberg D. M. (2011). Optimization of hapten-peptide labeling for pretargeted immunoPET of bispecific antibody using generator-produced 68Ga. J. Nucl. Med. 52, 555–559 10.2967/jnumed.110.083568 21421724

[B20] Knox S. J. Goris M. L. Tempero M. Weiden P. L. Gentner L. Breitz H. (2000). Phase II trial of yttrium-90-DOTA-biotin pretargeted by NR-LU-10 antibody/streptavidin in patients with metastatic colon cancer. Clin. Cancer Res. 6, 406–414 10690517

[B21] Kraeber-Bodéré F. Bardet S. Hoefnagel C. A. Vieira M. R. Vuillez J. P. Murat A. (1999a). Radioimmunotherapy in medullary thyroid cancer using bispecific antibody and iodine 131-labeled bivalent hapten: preliminary results of a phase I/II clinical trial. Clin. Cancer Res. 5, 3190s–3198s 10541363

[B22] Kraeber-Bodéré F. Faivre-Chauvet A. Saï-Maurel C. Campion L. Fiche M. Gautherot E. (1999b). Toxicity and efficacy of radioimmunotherapy in carcinoembryonic antigen-producing medullary thyroid cancer xenograft: comparison of iodine 131-labeled F(ab’)2 and pretargeted bivalent hapten and evaluation of repeated injections. Clin. Cancer Res. 5, 3183s–3189s 10541362

[B23] Kraeber-Bodéré F. Bodet-Milin C. Niaudet C. Saï-Maurel C. Moreau A. Faivre-Chauvet A. (2010). Comparative toxicity and efficacy of combined radioimmunotherapy and antiangiogenic therapy in carcinoembryonic antigen-expressing medullary thyroid cancer xenograft. J. Nucl. Med. 51, 624–631 10.2967/jnumed.109.070714 20351352

[B24] Kraeber-Bodéré F. Rousseau C. Bodet-Milin C. Ferrer L. Faivre-Chauvet A. Campion L. (2006). Targeting, toxicity, and efficacy of 2-step, pretargeted radioimmunotherapy using a chimeric bispecific antibody and 131I-labeled bivalent hapten in a phase I optimization clinical trial. J. Nucl. Med. 47, 247–255 16455630

[B25] Kraeber-Bodéré F. Saï-Maurel C. Campion L. Faivre-Chauvet A. Mirallié E. Chérel M. (2002). Enhanced antitumor activity of combined pretargeted radioimmunotherapy and paclitaxel in medullary thyroid cancer xenograft. Mol. Cancer Ther. 1, 267–274 12467222

[B26] Lam E. T. Ringel M. D. Kloos R. T. Prior T. W. Knopp M. V. Liang J. (2010). Phase II clinical trial of sorafenib in metastatic medullary thyroid cancer. J. Clin. Oncol. 28, 2323–2330 10.1200/JCO.2009.25.0068 20368568 PMC2881718

[B27] Laure Giraudet A. Al Ghulzan A. Aupérin A. Leboulleux S. Chehboun A. Troalen F. (2008). Progression of medullary thyroid carcinoma: assessment with calcitonin and carcinoembryonic antigen doubling times. Eur. J. Endocrinol. 158, 239–246 10.1530/EJE-07-0667 18230832

[B28] Le Doussal J. M. Chetanneau A. Gruaz-Guyon A. Martin M. Gautherot E. Lehur P. A. (1993). Bispecific monoclonal antibody-mediated targeting of an indium-111-labeled DTPA dimer to primary colorectal tumors: pharmacokinetics, biodistribution, scintigraphy and immune response. J. Nucl. Med. 34, 1662–1671 8410279

[B29] Liersch T. Meller J. Bittrich M. Kulle B. Becker H. Goldenberg D. M. (2007). Update of carcinoembryonic antigen radioimmunotherapy with (131)I-labetuzumab after salvage resection of colorectal liver metastases: comparison of outcome to a contemporaneous control group. Ann. Surg. Oncol. 14, 2577–2590 10.1245/s10434-006-9328-x 17570017

[B30] Liersch T. Meller J. Kulle B. Behr T. M. Markus P. Langer C. (2005). Phase II trial of carcinoembryonic antigen radioimmunotherapy with 131I-labetuzumab after salvage resection of colorectal metastases in the liver: five-year safety and efficacy results. J. Clin. Oncol. 23, 6763–6770 10.1200/JCO.2005.18.622 16170184

[B31] Liu G. Dou S. Baker S. Akalin A. Cheng D. Chen L. (2010). A preclinical 188Re tumor therapeutic investigation using MORF/cMORF pretargeting and an antiTAG-72 antibody CC49. Cancer Biol. Ther. 10, 767–774 10.4161/cbt.10.8.12879 21099368 PMC3093915

[B32] Liu G. Dou S. Liu Y. Wang Y. Rusckowski M. Hnatowich D. J. (2011). 90Y labeled phosphorodiamidate morpholino oligomer for pretargeting radiotherapy. Bioconjug. Chem. 22, 2539–2545 10.1021/bc200366t 21985267 PMC3244554

[B33] Liu G. Mangera K. Liu N. Gupta S. Rusckowski M. Hnatowich D. J. (2002). Tumor pretargeting in mice using (99m)Tc-labeled morpholino, a DNA analog. J. Nucl. Med. 43, 384–391 11884499

[B34] Machens A. Schneyer U. Holzhausen H.-J. Dralle H. (2005). Prospects of remission in medullary thyroid carcinoma according to basal calcitonin level. J. Clin. Endocrinol. Metab. 90, 2029–2034 10.1210/jc.2004-1836 15634717

[B35] McBride W. J. Sharkey R. M. Karacay H. D’Souza C. A. Rossi E. A. Laverman P. (2009). A novel method of 18F radiolabeling for PET. J. Nucl. Med. 50, 991–998 10.2967/jnumed.108.060418 19443594

[B36] McBride W. J. Zanzonico P. Sharkey R. M. Norén C. Karacay H. Rossi E. A. (2006). Bispecific antibody pretargeting PET (immunoPET) with an 124I-labeled hapten-peptide. J. Nucl. Med. 47, 1678–1688 17015905

[B37] Mirallié E. Vuillez J. P. Bardet S. Frampas E. Dupas B. Ferrer L. (2005). High frequency of bone/bone marrow involvement in advanced medullary thyroid cancer. J. Clin. Endocrinol. Metab. 90, 779–788 10.1210/jc.2004-1500 15572422

[B38] Morschhauser F. Radford J. Van Hoof A. Vitolo U. Soubeyran P. Tilly H. (2008). Phase III trial of consolidation therapy with yttrium-90-ibritumomab tiuxetan compared with no additional therapy after first remission in advanced follicular lymphoma. J. Clin. Oncol. 26, 5156–5164 10.1200/JCO.2008.17.2015 18854568

[B39] Paganelli G. Bartolomei M. Ferrari M. Cremonesi M. Broggi G. Maira G. (2001). Pre-targeted locoregional radioimmunotherapy with 90Y-biotin in glioma patients: phase I study and preliminary therapeutic results. Cancer Biother. Radiopharm. 16, 227–235 10.1089/10849780152389410 11471487

[B40] Paganelli G. Orecchia R. Jereczek-Fossa B. Grana C. Cremonesi M. De Braud F. (1998). Combined treatment of advanced oropharyngeal cancer with external radiotherapy and three-step radioimmunotherapy. Eur. J. Nucl. Med. 25, 1336–1339 10.1007/s002590050305 9724386

[B41] Peltier P. Curtet C. Chatal J. F. Le Doussal J. M. Daniel G. Aillet G. (1993). Radioimmunodetection of medullary thyroid cancer using a bispecific anti-CEA/anti-indium-DTPA antibody and an indium-111-labeled DTPA dimer. J. Nucl. Med. 34, 1267–1273 8326383

[B42] Rossi E. A. Goldenberg D. M. Cardillo T. M. McBride W. J. Sharkey R. M. Chang C.-H. (2006). Stably tethered multifunctional structures of defined composition made by the Dock and Lock method for use in cancer targeting. Proc. Natl. Acad. Sci. U.S.A. 103, 6841–6846 10.1073/pnas.0600982103 16636283 PMC1447525

[B43] Salaun P.-Y. Bodet-Milin C. Frampas E. Oudoux A. Saï-Maurel C. Faivre-Chauvet A. (2010). Toxicity and efficacy of combined radioimmunotherapy and bevacizumab in a mouse model of medullary thyroid carcinoma. Cancer 116, 1053–1058 10.1002/cncr.24792 20127950

[B44] Salaun P.-Y. Campion L. Bournaud C. Faivre-Chauvet A. Vuillez J.-P. Taieb D. (2012). Phase II trial of anticarcinoembryonic antigen pretargeted radioimmunotherapy in progressive metastatic medullary thyroid carcinoma: biomarker response and survival improvement. J. Nucl. Med. 53, 1185–1192 10.2967/jnumed.111.101865 22743249

[B45] Schlumberger M. J. Elisei R. Bastholt L. Wirth L. J. Martins R. G. Locati L. D. (2009). Phase II study of safety and efficacy of motesanib in patients with progressive or symptomatic, advanced or metastatic medullary thyroid cancer. J. Clin. Oncol. 27, 3794–3801 10.1200/JCO.2008.18.7815 19564535

[B46] Schoffelen R. van der Graaf W. T. A. Franssen G. Sharkey R. M. Goldenberg D. M. McBride W. J. (2010a). Pretargeted 177Lu radioimmunotherapy of carcinoembryonic antigen-expressing human colonic tumors in mice. J. Nucl. Med. 51, 1780–1787 10.2967/jnumed.110.079376 21051650

[B47] Schoffelen R. Sharkey R. M. Goldenberg D. M. Franssen G. McBride W. J. Rossi E. A. (2010b). Pretargeted immuno-positron emission tomography imaging of carcinoembryonic antigen-expressing tumors with a bispecific antibody and a 68Ga- and 18F-labeled hapten peptide in mice with human tumor xenografts. Mol. Cancer Ther. 9, 1019–1027 10.1158/1535-7163.MCT-09-0862 20354120 PMC2852483

[B48] Sharkey R. M. Goldenberg D. M. (2005). Perspectives on cancer therapy with radiolabeled monoclonal antibodies. J. Nucl. Med. 46(Suppl. 1), 115S–127S 15653660

[B49] Sharkey R. M. Rossi E. A. McBride W. J. Chang C.-H. Goldenberg D. M. (2010). Recombinant bispecific monoclonal antibodies prepared by the Dock-and-Lock strategy for pretargeted radioimmunotherapy. Semin. Nucl. Med. 40, 190–203 10.1053/j.semnuclmed.2009.12.002 20350628 PMC2855818

[B50] Shen S. Forero A. LoBuglio A. F. Breitz H. Khazaeli M. B. Fisher D. R. (2005). Patient-specific dosimetry of pretargeted radioimmunotherapy using CC49 fusion protein in patients with gastrointestinal malignancies. J. Nucl. Med. 46, 642–651 15809487

[B51] Tisell L. E. Oden A. Muth A. Altiparmak G. Mõlne J. Ahlman H. (2003). The Ki67 index a prognostic marker in medullary thyroid carcinoma. Br. J. Cancer 89, 2093–2097 10.1038/sj.bjc.6601453 14647143 PMC2376863

[B52] Tubiana M. Milhaud G. Coutris G. Lacour J. Parmentier C. Bok B. (1968). Medullary carcinoma and thyrocalcitonin. Br. Med. J. 4, 87–89 10.1136/bmj.4.5623.87 5748583 PMC1912102

[B53] Vuillez J. P. Peltier P. Caravel J. P. Chetanneau A. Saccavini J. C. Chatal J. F. (1992). Immunoscintigraphy using 111In-labeled F(ab’)2 fragments of anticarcinoembryonic antigen monoclonal antibody for detecting recurrences of medullary thyroid carcinoma. J. Clin. Endocrinol. Metab. 74, 157–163 10.1210/jc.74.1.157 1727816

[B54] Wells S. A. Jr. Gosnell J. E. Gagel R. F. Moley J. Pfister D. Sosa J. A. (2010). Vandetanib for the treatment of patients with locally advanced or metastatic hereditary medullary thyroid cancer. J. Clin. Oncol. 28, 767–772 10.1200/JCO.2009.23.6604 20065189 PMC2834392

[B55] Wells S. A. Jr. Robinson B. G. Gagel R. F. Dralle H. Fagin J. A. Santoro M. (2012). Vandetanib in patients with locally advanced or metastatic medullary thyroid cancer: a randomized, double-blind phase III trial. J. Clin. Oncol. 30, 134–141 10.1200/JCO.2011.35.5040 22025146 PMC3675689

